# Metabolic Dysfunction-Associated Steatotic Liver Disease Is Accompanied by Increased Activities of Superoxide Dismutase, Catalase, and Carbonyl Reductase 1 and Levels of miR-200b-3p in Mouse Models

**DOI:** 10.3390/antiox13111371

**Published:** 2024-11-09

**Authors:** Gabriela Svobodová, Michaela Šadibolová, Eva Velecká, Lucia Mráziková, Petra Vaculová, Petra Matoušková, Jaroslav Kuneš, Lenka Maletínská, Iva Boušová

**Affiliations:** 1 Department of Biochemical Sciences, Faculty of Pharmacy in Hradec Králové, Charles University, 50003 Hradec Králové, Czech Republic; vavrovaga@faf.cuni.cz (G.S.); sadibolm@faf.cuni.cz (M.Š.); veleckae@faf.cuni.cz (E.V.); matousp7@faf.cuni.cz (P.M.); 2 Institute of Organic Chemistry and Biochemistry, Czech Academy of Sciences, 16000 Prague, Czech Republic; lucia.mrazikova@uochb.cas.cz (L.M.); petra.vaculova@uochb.cas.cz (P.V.); jaroslav.kunes@fgu.cas.cz (J.K.); lenka.maletinska@uochb.cas.cz (L.M.); 3 Institute of Physiology, Czech Academy of Sciences, 14200 Prague, Czech Republic

**Keywords:** metabolic dysfunction-associated steatotic liver disease (MASLD), high fat, fructose, and cholesterol (FFC) diet, monosodium glutamate (MSG), detoxification enzymes, miRNAs, mice

## Abstract

Metabolic dysfunction-associated steatotic liver disease (MASLD), one of the leading causes of chronic liver disorders, is characterized by hepatic lipid accumulation. MASLD causes alterations in the antioxidant defense system, lipid, and drug metabolism, resulting in impaired antioxidant status, hepatic metabolic processes, and clearance of therapeutic drugs, respectively. In the MASLD pathogenesis, dysregulated epigenetic mechanisms (e.g., histone modifications, DNA methylation, microRNAs) play a substantial role. In this study, the development of MASLD was investigated in mice fed a high-fat, high-fructose, and high-cholesterol (FFC) diet from 2 months of age, mice treated neonatally with monosodium glutamate (MSG) on a standard diet (STD), and mice treated with MSG on an FFC diet at 7 months of age and compared to control mice (C) on STD. Changes in liver histology, detoxification enzymes, epigenetic regulation, and genes involved in lipid metabolism were characterized and compared. The strong liver steatosis was observed in MSG STD, C FFC, and MSG FFC, with significant fibrosis in the latter one. Moreover, substantial alterations in hepatic lipid metabolism, epigenetic regulatory factors, and expressions and activities of various detoxification enzymes (namely superoxide dismutase, catalase, and carbonyl reductase 1) were observed in MASLD mice compared to control mice. miR-200b-3p, highly significantly upregulated in both FFC groups, could be considered as a potential diagnostic marker of MASLD. The MSG mice fed FFC seem to be a suitable model of MASLD characterized by both liver steatosis and fibrosis and substantial metabolic dysregulation.

## 1. Introduction

Chronic liver disease is a major health problem worldwide. One of the leading causes in developed countries is metabolic dysfunction-associated steatotic liver disease (MASLD), previously referred to as non-alcoholic fatty liver disease, which affects a quarter of the global adult population [1]. MASLD is an umbrella term that covers a broad clinical spectrum of liver abnormalities varying in the severity of the injury and resulting fibrosis. Steatotic liver disease (hepatic steatosis, SLD) at one end of the spectrum may progress to a more advanced stage called metabolic dysfunction-associated steatohepatitis (MASH), with significant hepatocyte damage, inflammation, and pericellular fibrosis. About 3–5% of biopsy-proven MASH patients were shown to progress to cirrhosis, liver failure, and hepatocellular carcinoma [2,3]. A combination of genetic and environmental factors contributes to the development of MASLD. The components of metabolic syndrome, such as obesity, insulin resistance, and type 2 diabetes mellitus, sedentary lifestyle, and Western diet, belong to the risk factors associated with MASLD [4].

Various animal models mimicking MASLD/MASH have been developed to elucidate the progression of MASLD to MASH and its link to metabolic syndrome [3,5]. Obesogenic dietary models imitate overnutrition and a sedentary lifestyle leading to obesity similar to that in humans; therefore, the use of diet-induced obesity (DIO) models simulates the natural development of MASLD. Particularly, a combination of a high-fat diet with a fructose/sucrose and cholesterol (FFC) diet leads to the rapid development of MASLD in mice. These mouse models greatly mimic features of human obesity and steatosis, fibrosis, and inflammation. Feeding mice with the FFC diet results in increased body and liver weight, plasma cholesterol, alanine aminotransferase, aspartate aminotransferase, and hepatic triacylglycerols (TAGs) as well as increased proinflammatory cytokines, galectin-3, and other MASLD markers [6,7,8]. Homeostatic brain areas responsible for regulating food intake and energy homeostasis are located in the hypothalamus. The arcuate nucleus (ARC) of the hypothalamus is the target of circulating energy homeostatic peptides, including leptin, insulin, and ghrelin. Insulin and leptin activate their respective receptors in the hypothalamus, a key brain area for transmitting peripheral weight-regulating signals to the brain. Early-onset obesity could be induced by subcutaneous administration of monosodium glutamate (MSG) to newborn mice. This administration causes damage to the ARC and disrupts leptin, ghrelin, and insulin signaling in this region, resulting in elevated levels of leptin and insulin [9]. Impaired ghrelin signaling results in deficient growth hormone secretion and resulting in low serum levels of insulin-growth factor 1. Early infant growth hormone deficiency and hyperinsulinemia are thought to predispose late-in-life chronic conditions [10]. MSG-obese rodents develop obesity with increased adiposity while maintaining their body weight, primarily due to a lower metabolic rate rather than increased food consumption. The presence of hyperinsulinemia alongside nearly normal blood glucose levels supports the utility of MSG-obese mice as a model for pre-diabetes [11,12,13]. Coelho et al. found that MASLD is a precocious event in outbred Swiss mice with MSG-induced obesity [14].

As the pathogenesis of MASLD is a complex and multifactorial process, it is yet not fully understood. Metabolic changes leading to fat accumulation in hepatocytes (steatosis) include increased uptake of circulating free fatty acids (FFAs) or de novo lipogenesis and decreased fatty acid oxidation or lipoprotein export [15]. Lipidomic profiling showed significantly elevated levels of diacylglycerols, TAGs, and free cholesterol and decreased content of phosphatidylcholine and unsaturated fatty acids in the livers of MASLD/MASH patients compared to healthy subjects [16]. Indeed, alterations in the expression of enzymes involved in hepatic lipid metabolism and in signaling pathways regulating lipid metabolism were reported in MASLD [17,18]. Moreover, intrahepatic lipid accumulation leads to metabolic disturbances resulting in excessive mitochondrial production of reactive oxygen species (ROS) [19]. Oxidative stress contributes to the progression of SLD to a more severe form of MASH [20] and promotes activation of cellular antioxidant mechanisms (antioxidant enzymes and non-enzymatic antioxidants) during MASLD that counteract ROS production [21,22]. The severity of changes in the antioxidant system may also be related to the stage of the disease [23].

Given the close association of MASLD with metabolic syndrome and the increased risk of cardiovascular diseases, many MASLD patients are prescribed a variety of medications to manage these associated conditions [24]. A major part of drug metabolism is carried out in the liver, undergoing significant structural and pathophysiological changes during MASLD, which are likely to influence drug-metabolizing enzymes (DMEs) [25,26,27]. Dysregulations of DMEs may result in altered drug metabolism, causing a generation of toxic metabolites, and changes in the bioavailability and pharmacotherapeutic effects of drugs [28,29].

Epigenetic modifications, heritable but reversible modifications in gene expression not involving changes in DNA sequence, comprising DNA methylation, histone modifications, and non-coding RNA-mediated gene regulation, may contribute to disease risk [30,31]. MicroRNAs (miRNAs), a type of non-coding RNAs, regulate gene expression at the post-transcriptional level and are among the frequently studied candidate biomarkers of MASLD. Several miRNAs have been shown to participate in the pathogenesis of MASLD and type 2 diabetes mellitus, in which aberrant miRNA expression patterns have been reported [32,33]. Circulating miRNA signature, which mirrors the extent of liver injury and correlates with fibrosis staging, could be a suitable non-invasive biomarker of MASLD [34,35]. Moreover, miRNA-based therapeutic strategies for MASLD/MASH are currently being developed [36]. Indeed, altered expression of some epigenetic regulatory enzymes (e.g., DNA-methyltransferase 1, sirtuin 1, histone acetyltransferase 1) has been reported in both SLD and MASH stages [31,37,38]. These alterations likely contribute to the dysregulation of lipid metabolism, inflammation, and cell death described in MASLD [31]. Furthermore, functionally relevant changes in DNA methylation could distinguish between patients with advanced vs. mild stages of MASLD [39].

The objective of this study was to investigate and compare the development of MASLD in mice on an FFC diet, mice treated neonatally with MSG on a standard diet (STD), and mice treated with MSG on an FFC diet. As controls, mice without MSG treatment on STD were used; all mice were of NMRI strain. We focused on the connections among obesity, inflammation, disrupted lipid and glucose metabolism, and oxidative stress during the progression of MASLD. Furthermore, the study aimed to describe alterations in DMEs and epigenetic factors and to propose potential markers that could be employed in future pharmacological investigations focused on MASLD progression.

## 2. Materials and Methods

### 2.1. Animals and Experimental Design

NMRI mice obtained from Charles River (Sulzfeld, Germany) were housed in a regular animal facility (6 per cage) in a temperature- and light-controlled environment at 22 °C with a 12 h light/dark cycle and free access to tap water and a standard chow diet. The animals were handled in accordance with good animal practices according to the ethical guidelines stated in the Guide for the Care and Use of Laboratory Animals (Protection of Animals from Cruelty Act 246/92, Czech Republic). All animal experimental procedures were approved by the Committee for Experiments with Laboratory Animals of the Czech Academy of Sciences (permit No. 96/2020).

**Figure 1:**
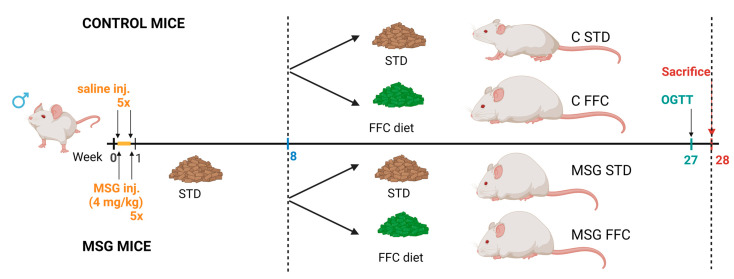
Schematic representation of experimental design and timeline. The figure was created with biorender.com (accessed on 22 October 2024).

Newborn male NMRI mice were divided into two groups as follows: Control mice and mice with monosodium glutamate (MSG)-induced obesity. For hypothalamic lesion-induced obesity, MSG (4 mg/g of body weight) was subcutaneously injected into newborn mice daily from postnatal day 2 to 5 as described previously [13]. Controls were injected with saline of osmolality corresponding to the MSG solution. At the age of 2 months, mice were split into 4 groups of 6 mice per group: the first group (C STD) consisted of control mice fed a standard rodent chow diet (STD) Ssniff R/M-H (Ssniff Spezialdiaten GmbH, Soest, Germany) containing 33% protein, 9% fat, and 58% carbohydrates; the second group (C FFC) consisted of control mice fed an FFC diet (Research diets—D16010101, New Brunswick, NJ, USA) containing 40% kcal from fat, 22% kcal from fructose, and 1.8% kcal from cholesterol; and mice with MSG-induced obesity were exposed to either a STD or FFC diet belonging to the third (MSG STD) and fourth (MSG FFC) group, respectively. All groups were fed ad libitum for a 20-week-long period. During this 20-week-long period, the body weight was measured once per week. At the age of 27 weeks, one week before the end of the experiment, mice fasted for 6 h, and an oral glucose tolerance test (OGTT) was performed. At the age of 28 weeks, the mice were fasted overnight, blood plasma samples were collected from the tail veins for determination of the biochemical parameters (levels of TAGs, FFAs, cholesterol, insulin, and leptin), and then the animals were deeply anesthetized with pentobarbital (170 mg/kg of BW, Sigma Aldrich, Prague, Czech Republich) and transcardially perfused with ice-cold saline solution supplemented with heparin (10 U/mL, Zentiva, Prague, Czech Republic). The experimental design and timeline are depicted in Figure 1. Tissue samples, i.e., gonadal white adipose tissue (gWAT) and liver, were dissected, weighed, and stored at −80 °C until use. The caudate lobes of each liver were used for liver histology. The obesity rate was expressed by the Lee index, defined as the cube root of body mass (g) divided by nasoanal length (NA) (in cm) [40].

### 2.2. Oral Glucose Tolerance Test

OGTT was performed after 6 h of fasting. At time point 0, glucose was determined in blood drawn from the tail vessels, and a glucose solution at a dose of 2 g/kg of BW was loaded perorally by gavage. The blood glucose concentration was determined in whole blood at 15, 30, 60, 120, and 180 min using a glucometer (Arkray, Tokyo, Japan).

### 2.3. Liver Histological Evaluation

The main histological characteristic of MASLD is the intracellular lipid accumulation in hepatocytes. To confirm this phenotype, we followed our previously described routine histological procedures [41]. Briefly, the caudal lobe of each liver was fixed with 4% paraformaldehyde, embedded into paraffin blocks, and cut on a microtome (Leica RM2255, Leica Biosystems, Buffalo Grove, IL, USA) into 5 µm thickness sections. After deparaffinization in xylene and rehydration in an ethanol gradient, sections were stained in Weigert’s iron hematoxylin solution (MilliporeSigma, Burlington, MA, USA) and eosin Y (Carl Roth GmbH + Co., Karlsruhe, Germany) for the determination of fat droplets [42], and in Picrosirius red for the determination of collagen fibers before being examined under a light microscope (Olympus IX83, Olympus Europa SE & Co. KG, Hamburg, Germany) at 200× magnification.

### 2.4. Biochemical Parameters Determination

The colorimetric assays were used to measure plasmatic levels of FFAs (Roche, Mannheim, Germany) as well as plasmatic levels of TAGs and cholesterol (Erba Lachema, Brno, Czech Republic). The plasma insulin concentrations were measured using an RIA assay (Millipore, St. Charles, MI, USA), and the leptin concentrations were determined by ELISA (Millipore, St. Charles, MI, USA). The plasma aspartate aminotransferase (AST) activity was assessed using a colorimetric assay kit (Sigma-Aldrich, Prague, Czech Republic). The fluorometric assay kit was used to determine hepatic TAG content (Sigma-Aldrich, Prague, Czech Republic). All measurements were performed according to the manufacturer’s instructions.

### 2.5. Tissue RNA Extraction and cDNA Synthesis

Total RNA was extracted using TriReagent according to the manufacturer’s instructions (Biotech, Prague, Czech Republic). The homogenization of each sample was performed with a HG-24 homogenizer (Biobase, Jinan, China) for two 20 s intervals with a speed of 7.0 m/s. The concentration as well as purity of the total RNA content in each sample were determined spectrophotometrically using the SPARK^®^ multimode microplate reader (Tecan Group, Mannedorf, Switzerland). To estimate the RNA integrity, agarose gel electrophoresis on RNA Nano chips was performed using an Agilent 2100 bioanalyzer (Agilent Technologies, Santa Clara, CA, USA), and the values of the RNA integrity number (RIN) described the quality of RNA.

To avoid genomic DNA contamination, 10 µg of isolated RNA were treated with DNase I (New England Biolabs, Ipswich, MA, USA) following the manufacturer’s instructions. The RNA at a final concentration of 0.2 µg/µL was subsequently used for reverse transcription using ProtoScript II reverse transcriptase (New England Biolabs, Ipswich, MA, USA) and random hexamers or specific oligonucleotide STEM-Loop miRNA RT primers (Generi Biotech, Hradec Králové, Czech Republic) under the conditions recommended by the supplier [43]. Afterward, the obtained cDNAs were diluted 1:9 with double distilled water (ddH_2_O) and stored at −20 °C until the qPCR assays.

### 2.6. Quantitative Polymerase Chain Reaction (qPCR)

Determination of the mRNA expression of genes of interest (superoxide dismutase 1 (*Sod1*), catalase (*Cat*), glutathione peroxidase 4 and 7 (*Gpx4*, *Gpx7*), glutathione reductase (*Gr*), glutathione S-transferase M1, M3, and P (*Gstm1*, *Gstm3*, *Gstp1*), NAD(P)H:quinone oxidoreductase 1 (*Nqo1*), cytochrome P450 1A1/2 (*Cyp1a1/2*), aldo-keto reductase 1C6 and 1C20 (*Akr1c6*, *Akr1c20*), carbonyl reductase 1 (*Cbr1*), DNA-methyltransferase 1 and 3A (*Dnmt1*, *Dnmt3a*), histone deacetylase 1–6 and 11 (*Hdac1*-*Hdac6*, *Hdac11*), sirtuin 1–3 (*Sirt1*-*Sirt3*), histone acetyltransferase 1 (*Hat1*), lysine acetyltransferase 2B (*Kat2b*), E1A binding protein p300 (*Ep300*), peroxisome proliferator-activated receptor alfa and gamma (*Ppar*-α, *Ppar-γ*), acetyl-CoA carboxylase alpha (*Acaca*), fatty acid synthase (*Fasn*), stearoyl-coenzyme A desaturase 1 and 2 (*Scd1*, *Scd2*), sterol regulatory element binding transcription factor 2 (*Srebf2*), 3-hydroxy-3-methylglutaryl-CoA reductase (*Hmgcr*), glycerol-3-phosphate acyltransferase mitochondrial (*Gpam*), cell death inducing DFFA like effector c (*Cidec*), perilipin 2 and 5 (*Plin2*, *Plin5*)) and miRNA expression (miR-16-5p, miR-21-5p, miR-29b-3p, miR-33a-5p, miR-122-3p, miR-122-5p, miR-152-3p, miR-200a-3p, miR-200b-3p, miR-221-3p, miR-335-5p, and miR-451a) were carried out in the QuantStudio™ 6 Flex Real-Time PCR System (Applied Biosystems, Foster City, CA, USA) using the Xceed SYBR Green I detection (Institute of Applied Biotechnology, Prague, Czech Republic) in a final volume of 20 µL. The reaction mixture consisted of components from the qPCR Core kit for SYBR Green I as specified by the manufacturer, both specific forward primers and specific reverse primers or universal reverse primers, and 2 µL of diluted cDNA corresponding to 10 ng of reverse-transcribed mRNA or miRNA, respectively. All primers were designed manually and synthesized by Generi Biotech (Hradec Králové, Czech Republic). The sequences of all qPCR primers as well as gene accession numbers of all genes are shown in Table S1. The qPCR efficiency of each primer pair was verified in our experimental set-up as was reported previously [44]. The qPCR reactions followed previously described thermocycling profiles [45], with fluorescence data acquired at the end of each amplification step. A melting curve protocol [46] served to investigate the specificity of the qPCR reaction. Relative mRNA expression levels of the target genes were expressed as fold changes in the treated groups relative to the C STD group using the 2^−ΔΔCt^ method [47]. After verification of the stability in our experimental set-up, *Gapdh* (glyceraldehyde 3-phosphate dehydrogenase), *Rplp0* (ribosomal protein, large, P0), and miR-93-5p were used as reference genes for the mRNA and miRNA expression normalization, respectively.

### 2.7. Western Blotting

Protein expression of the enzymes of interest (SOD1, CAT, GPx4, GPx7, GR, GSTM, GSTP1, CYP1A1/2, AKR1C, CBR1, and NQO1) and collagen type I chain α1 (COL1A1) was performed using the Western blotting technique. Approximately 100 mg of liver tissue, preserved in 300 µL of lysis buffer, underwent homogenization using a Bullet Blender homogenizer (Next Advance, Inc., Averill Park, NY, USA), followed by sonication for 1 min. The protein concentration in each liver supernatant sample was quantified using the bicinchoninic acid (BCA) assay as described by the manufacturer (Sigma Aldrich, Prague, Czech Republic). Subsequently, 20 µg of the proteins were loaded onto 4–15% Criterion™ TGX™ precast gels (Bio-Rad, Hercules, CA, USA). SDS-PAGE electrophoresis, transferring the separated proteins onto nitrocellulose membranes using the Criterion™ blotter (Bio-Rad, Hercules, CA, USA), and subsequent overnight blocking in 5% non-fat dried milk were followed by membrane incubation with the respective primary antibodies and horseradish peroxidase-conjugated secondary antibodies (Table S2) for 1 h at room temperature. For protein band visualization, a chemiluminescence kit (GE Healthcare, Buckinghamshire, UK) was employed, and image acquisition along with band intensity evaluation were performed using the Alliance Q9 Advanced Chemiluminescence Imager (Uvitec, Cambridge, UK). To quantify relative protein expression, protein signal intensities were normalized with respect to the signal intensities of β-actin (ACTB) or GAPDH (for quantification of COL1A1), serving as the internal loading controls.

### 2.8. Preparation of Subcellular Fractions

Liver tissue was weighed and homogenized in an ice-cold 0.1 M sodium phosphate buffer (pH 7.4) at a 1:6 ratio using a Potter-Elvehjem homogenizer (bbi-biotech, Berlin, Germany) and sonication with Sonopuls (Bandelin, Berlin, Germany). The 20,000× *g* supernatant was obtained by centrifuging the liver homogenate at 20,000× *g* for 65 min at 4 °C, and the protein concentration was quantified by the BCA method following the manufacturer’s protocol (Sigma Aldrich, Prague, Czech Republic). The 20,000× *g* supernatant was then stored at −80 °C until further analyses.

### 2.9. Enzyme Assays

Specific or total activities of selected enzymes were determined spectrophotometrically at specific wavelengths, detecting either the formation of products or the decrease in substrate, product, or cofactor levels using the Tecan Infinite M200PRO or SPARK^®^ multimode microplate reader (Tecan Group, Mannedorf, Switzerland), respectively. Sample dilution was chosen within the linear phase of the reaction, and the final reaction mixtures contained organic solvents not exceeding 1% (*v*/*v*). To quantify enzyme catalytic activities, the obtained data were normalized to milligrams (mg) of protein.

The specific activity of SOD1 was determined indirectly using the SOD Determination Kit-WST following the instructions specified by the manufacturer (Sigma Aldrich, Prague, Czech Republic). To assess the specific activity of CAT, a slightly modified method based on the reaction of ammonium molybdate with H_2_O_2_ was employed [48]. Oxidized glutathione (GSSG) and *t*-butyl hydroperoxide were used as specific substrates for the determination of GR-specific activity and GPx total catalytic activity, respectively [49,50]. The overall GST activity was assessed by a slightly modified standard colorimetric assay using glutathione (GSH) and 1-chloro-2,4-dinitrobenzene (CDNB) as an electrophilic substrate [51].

The fluorescence emitted by the formed resorufin in a CYP1A1/2-catalyzed reaction served for the quantification of its specific activity [52]. The specific activities of carbonyl-reducing enzymes (AKR1C and CBR1) were assessed using previously reported methods [53,54] with slight modifications. The increase in absorbance of reduced cytochrome c was used for the determination of the NQO1-specific activity [55].

### 2.10. Statistical Analysis

Statistical analysis and the generation of all the graphs in this paper were conducted using GraphPad Prism software version 9.0 (GraphPad Software, San Diego, CA, USA). Gene expression results presented in this study are derived from six independent mouse liver samples per group as biological replicates, each with duplicates as technical replicates. Similarly, protein expression results are based on six independent mouse liver samples per group as biological replicates, and catalytic activity results are derived from three independent experiments of each from mouse liver samples pooled from all groups (n = 6), each with duplicates to tetraplicates as technical replicates, respectively. Data are expressed as mean ± standard deviation (SD). Differences between groups were analyzed using one-way ANOVA with Bonferroni’s post hoc test. The *p*-values < 0.05 were considered to indicate the statistical significance.

## 3. Results

### 3.1. FFC and MSG Alters the Body Weight, Liver Weight, and Plasma Biochemical Parameters

The NMRI strain was chosen in this study based on our previous study [56], in which the MSG mice of both sexes, of the NMRI and C57BL/6 strains, were compared regarding their daily food intake, body weight, fat, and liver weight. MSG mice of both strains and both sexes had very substantially increased fat weight compared to controls. The most striking difference between the strains was that NMRI males had significantly higher fasting glucose and insulin levels, resulting in a 28-times higher HOMA (Homeostasis Model Assessment—Insulin Resistance) index than their controls, while MSG C57BL/6 males did not differ from controls in fasting glucose and insulin levels and HOMA index. Even though NMRI is an outbred strain and C57BL/6 is an inbred strain, the SEM of morphometric and metabolic parameters did not differ substantially. Moreover, NMRI pups are more robust, and nearly all survive repeated MSG treatment at an early age.

**Figure 2:**
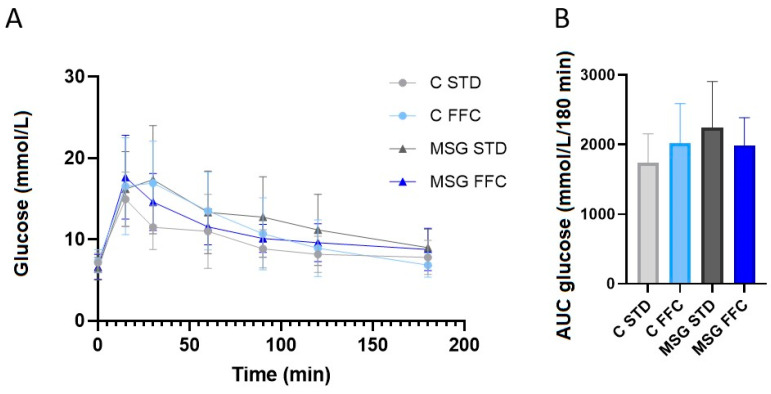
Oral glucose tolerance test (OGTT) measured in controls on STD and FFC diets and in MSG on STD and FFC diets at the end of the experiment. (**A**) Blood glucose during OGTT; (**B**) OGTT results expressed as the AUC of glucose at 180 min after ingestion. Data are presented as means ± SD. Statistical analysis was performed by two-way ANOVA with Bonferroni´s post hoc test.

Both C and MSG mice on the FFC diet were significantly heavier than their respective controls at the age of 7 months, while there were no significant differences between the controls and the MSG group fed with STD. MSG-treated mice had shorter nasoanal (NA) lengths compared to the controls; however, the gWAT weight of the MSG mice on STD was significantly higher than in control mice on STD. The Lee index of obesity differed in controls and the MSG mice both on STD. The liver weight of both C and MSG mice fed the FFC diet was significantly higher than in the respective control groups. OGTT did not show significant changes to the tolerance to glucose, neither between controls and MSG mice nor between groups with different diets (Figure 2). Regarding biochemical parameters, no difference in the plasma TAG levels, FFA levels, or insulin concentration was observed among the experimental groups. Plasma cholesterol levels were found to be elevated in C FFC and MSG FFC groups of mice. Plasmatic levels of AST, a marker of hepatocellular injury, tended to increase in both FFC diet-fed groups. As MSG application causes disruption of leptin signaling [57], the plasma leptin concentration was evaluated. As expected, plasma leptin levels were markedly higher in all MSG-treated groups than in corresponding controls. All data are summarized in Table 1.

### 3.2. Histological Evaluation of the Liver

**Figure 3:**
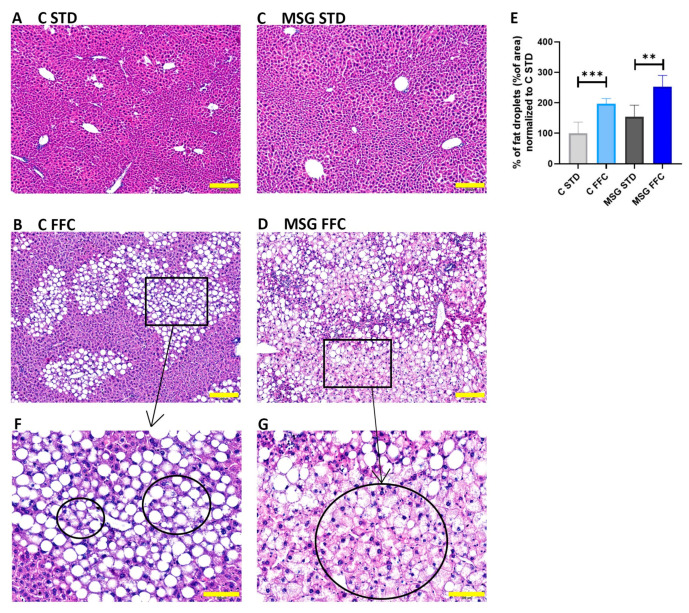
Histological examination of liver sections stained in hematoxylin/eosin. Representative photomicrographs of hematoxylin/eosin staining. (**A**) Quantification of liver steatosis represented by % of fat droplets showed normal liver tissue without infiltration of fat droplets in C STD mice; (**B**) mild infiltration of fat droplets represented by macro-vesicular liver steatosis in C FFC mice; (**C**) extensive infiltration of fat droplets represented by micro-vesicular liver steatosis in MSG STD mice; (**D**) and very extensive infiltration of fat droplets represented by a combination of macro- and micro-vesicular liver steatosis in MSG FFC mice. The photomicrographs are shown at a magnification of 200×, scale bar 200 µm (**A**–**D**). (**E**) Quantification of liver steatosis is represented as % of fat droplets. Results are presented as the mean ± SD (n = 6), with C STD set to 100%. Statistical analyses were performed using one-way ANOVA with Bonferroni´s post hoc test. The *p*-value thresholds were defined as ** (*p* < 0.01) and *** (*p* < 0.001). (**F**) Hepatocyte ballooning (circle) in the liver of C FFC mice. (**G**) Hepatocyte ballooning (circle) in the liver of MSG FFC mice. Scale bar 100 µm for (**F**,**G**). Box represents magnified area presented in (**F**,**G**).

The presence of steatosis in hepatocytes was evaluated microscopically in liver sections stained with hematoxylin and eosin. Histological examination of liver slices displayed strong liver steatosis in the liver of mice fed the FFC diet in both C FFC and MSG FFC groups of mice compared to their respective controls, as shown by the massive accumulation of fat droplets (Figure 3B,D). Also, extensive steatosis was observed in the MSG STD group compared to the C STD group (Figure 3A,C). The increased fat content in the liver of mice fed the FFC diet in both C FFC and MSG FFC groups of mice compared to their respective controls was confirmed by the hepatic TAG content determination (Table 1). Hepatocellular ballooning, a key histological feature of MASH, was observed in both FFC diet-fed groups (Figure 3F,G).

**Figure 4:**
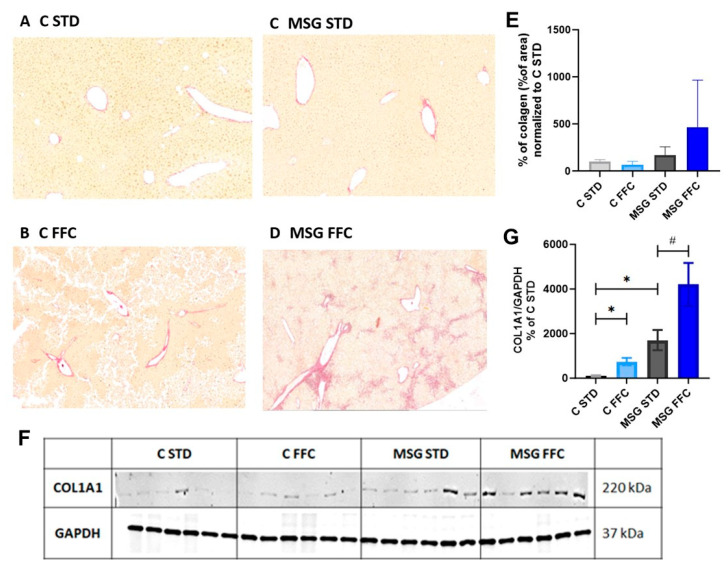
Histological examination of liver sections stained in Picrosirius red and impact of the FFC diet on producing COL1A1 in the liver. Representative photomicrographs of Picrosirius Red staining of (**A**) hepatic collagen in C STD mice, (**B**) C FFC mice, (**C**) MSG STD mice, and (**D**) in MSG FFC mice. The photomicrographs are shown at a magnification of 200×. (**E**) Quantification of liver fibrosis is represented as % of hepatic collagen. Results are presented as the mean ± SD (n = 6), with C STD set to 100%. Statistical analyses were performed using one-way ANOVA with Bonferroni´s post hoc test. (**F**) Representative Western blot images of COL1A1. (**G**) Relative quantification of COL1A1 protein expression is represented as % of C STD. Results are presented as the mean ± S.E.M. (n = 6), with C STD set to 100%. Statistical analyses were performed using one-way ANOVA with Bonferroni´s post hoc test. The *p*-value thresholds were defined as * (*p* < 0.05) compared to the C STD group and as # (*p* < 0.05) compared to the MSG STD group, respectively.

Hepatic fibrosis was assessed through Picrosirius Red staining of hepatic collagens in the MSG FFC group compared to the MSG STD group, while the C FFC group did not show increased fibrosis compared to the C STD group (Figure 4A–E). Western blot analysis of COL1A1 revealed enhanced collagenous matrix deposition in the liver of MSG mice fed the STD as well as MSG and control mice fed the FFC diet (Figure 4F,G).

### 3.3. FFC Diet and MSG Application Affect Activity and Expression of Antioxidant Enzymes

Enzymatic activities of several antioxidant enzymes (SOD, CAT, GPx, GR, and GST) were assayed using specific substrates in 20,000× *g* supernatant of pooled samples obtained from mouse liver, whereas corresponding levels of mRNA and protein were measured in samples from individual animals (n = 6).

**Figure 5:**
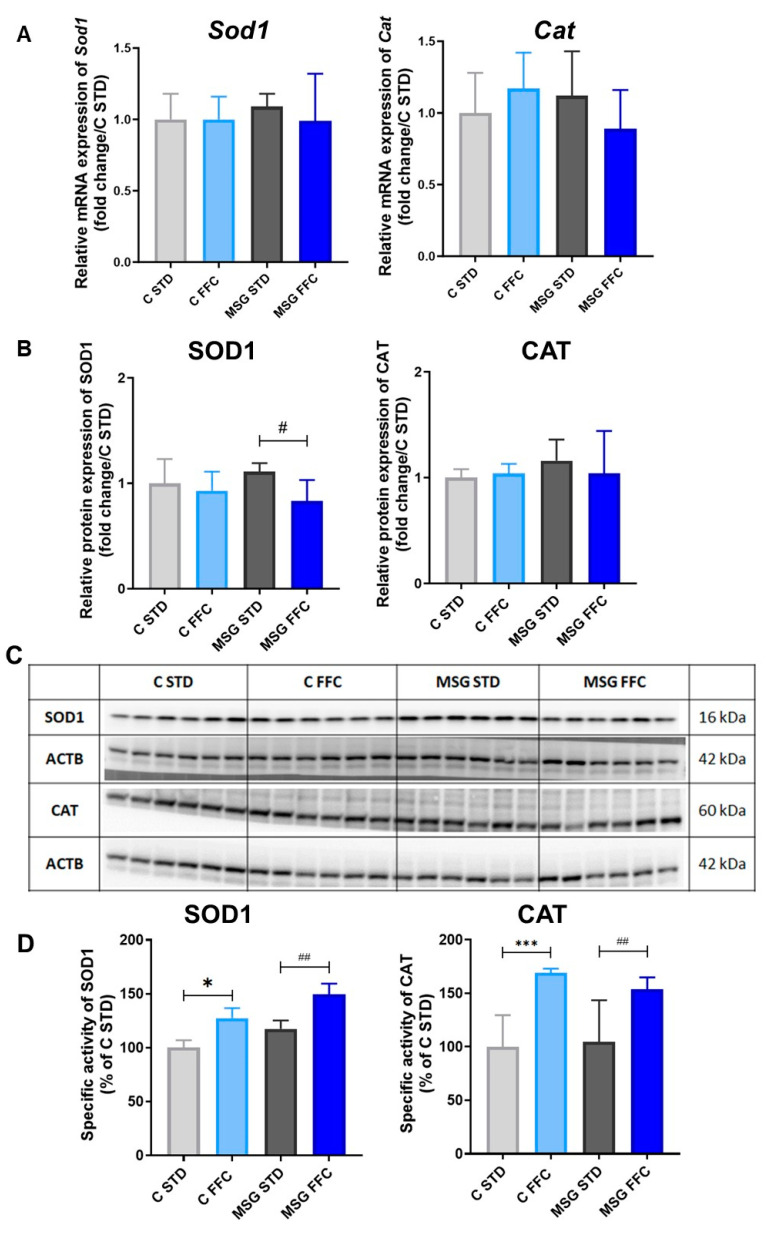
Superoxide dismutase and catalase expression and activity in the liver of MASLD models. (**A**) Relative quantification of *Sod1* and *Cat* mRNA expression. (**B**) Relative protein expression of SOD1 and CAT. (**C**) Representative Western blot images of SOD1 and CAT. (**D**) Relative quantification of specific activities of SOD1 and CAT. Statistical analyses were performed using one-way ANOVA with Bonferroni’s post hoc test. The *p*-value thresholds were defined as * (*p* < 0.05) and *** (*p* < 0.001) compared to the C STD group and as # (*p* < 0.05) and ## (*p* < 0.01) compared to the MSG STD group, respectively.

Feeding with the FFC diet caused an increase in SOD1 activity both in C and MSG mice compared to their respective controls (Figure 5D). The mRNA and protein quantity of SOD1 did not differ among the studied groups (Figure 5A–C). Even though the CAT mRNA and protein expressions were unchanged (Figure 5A–C), activity was higher in both groups fed the FFC diet, while no changes were found among the C STD and MSG STD groups (Figure 5D). Activity as well as mRNA level of GR remained unchanged regardless of the obesity induction method or type of diet (Figure S1).

**Figure 6:**
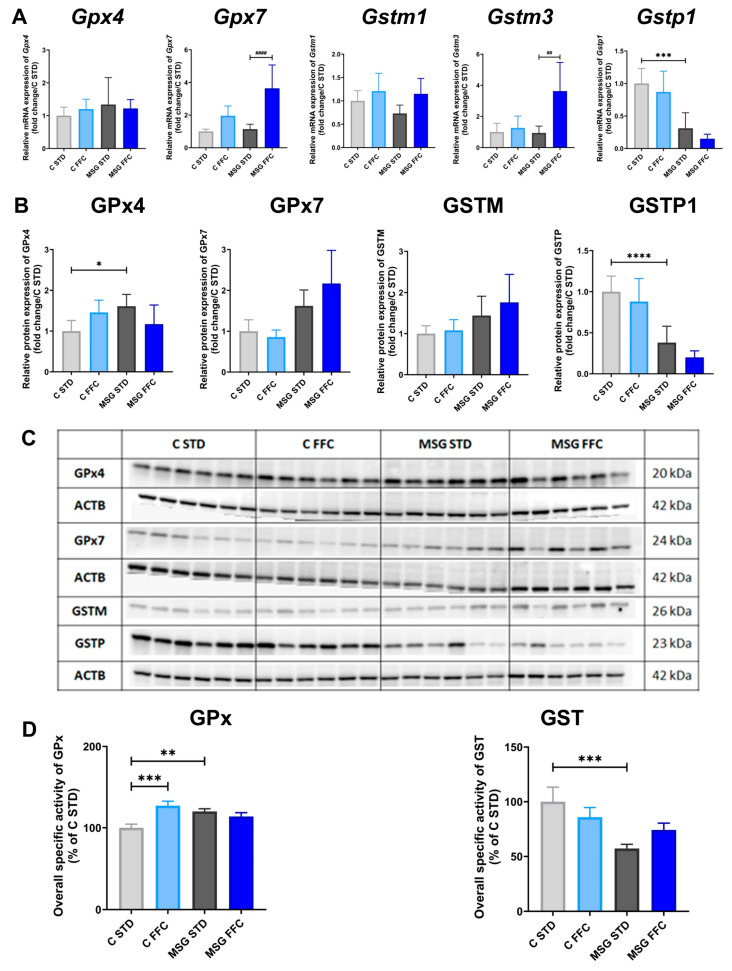
Glutathione peroxidase and glutathione S-transferase expression and activity in the liver of MASLD models. (**A**) Relative quantification of *Gpx4*, *Gpx7*, *Gstm1*, *Gstm3*, and *Gstp1* mRNA expression. (**B**) Relative protein expression of GPx4, GPx7, GSTM, and GSTP. (**C**) Representative Western blot images of GPx4, GPx7, GSTM, and GSTP. (**D**) Relative quantification of specific activities of GPx and GST. Statistical analyses were performed using one-way ANOVA with Bonferroni’s post hoc test. The *p*-value thresholds were defined as * (*p* < 0.05), ** (*p* < 0.01), *** (*p* < 0.001), and **** (*p* < 0.0001) compared to the C STD group, and as ## (*p* < 0.01) and #### (*p* < 0.0001) compared to the MSG STD group, respectively.

Regarding GPx, overall enzymatic activity was higher in MSG than in control mice, and its activity significantly rose in control mice on the FFC diet (Figure 6D). In mice, GPx comprises eight isoforms [58]; in this study, we tested the mRNA levels of *Gpx4* and *Gpx7*. The expression of the most abundant isoform GPx1, which reacts with H_2_O_2_ and small soluble hydroperoxides but is less effective against complex lipid hydroperoxides, is modulated by selenium levels [58]. A GPx1 deficiency can worsen cardiac dysfunction, while its overexpression or increased activity has been linked to poor prognosis in various cancers [59]. The GPx3, a major ROS scavenger in plasma neutralizing complex hydroperoxides, is also an established biomarker of selenium status [60]. In hepatocellular carcinoma, almost 80% of tumor samples exhibited GPx3 hypermethylation, leading to its markedly reduced mRNA and protein levels, suggesting its potential as a broad-spectrum tumor screening marker due to its measurable presence in plasma [61]. The GPx4, a membrane-associated phospholipid hydroperoxidase ubiquitously expressed in tissues, is the only GPx isoform that directly reduces and detoxifies lipid hydroperoxides [58]. The GPx7 is an essential intracellular sensor for oxidative stress and endoplasmic reticulum (ER) stress [62,63], which is involved in oxidative protein folding in ER [64].

The mRNA levels of *Gpx7* increased in MSG mice fed with the FFC diet (Figure 6A), while its protein levels remained unchanged in all groups (Figure 6B,C). Protein levels of GPx4 were higher in MSG STD compared to C STD mice (Figure 6B,C), while its mRNA levels remained unchanged (Figure 6A). The total activity of hepatic GSTs, measured using universal substrate CDNB, was lower in MSG mice than in control ones (Figure 6D). Immunoblotting using class-specific polyclonal antibodies showed changes in protein expression of M and P GST classes. In MSG mice, a significantly lower P-class GST protein level was found, while that of M-class GST was slightly increased. No significant effect of the FFC diet was observed (Figure 6B,C). In mice, cytosolic GSTs are encoded by 16 genes sub-divided into seven classes. In this study, mRNA levels of three isoforms were measured. The mRNA levels of *Gstp1* were significantly lower in MSG mice; FFC diet-fed MSG mice had upregulated *Gstm3* mRNA levels (Figure 6A).

### 3.4. FFC and MSG Causes Changes in Expression and Activity of Biotransformation Enzymes

The effect of MASLD on the specific activities, protein, and mRNA expressions of several Phase I biotransformation enzymes were tested (CYP1A1/2, CBR1, NQO1, and AKR1C).

**Figure 7:**
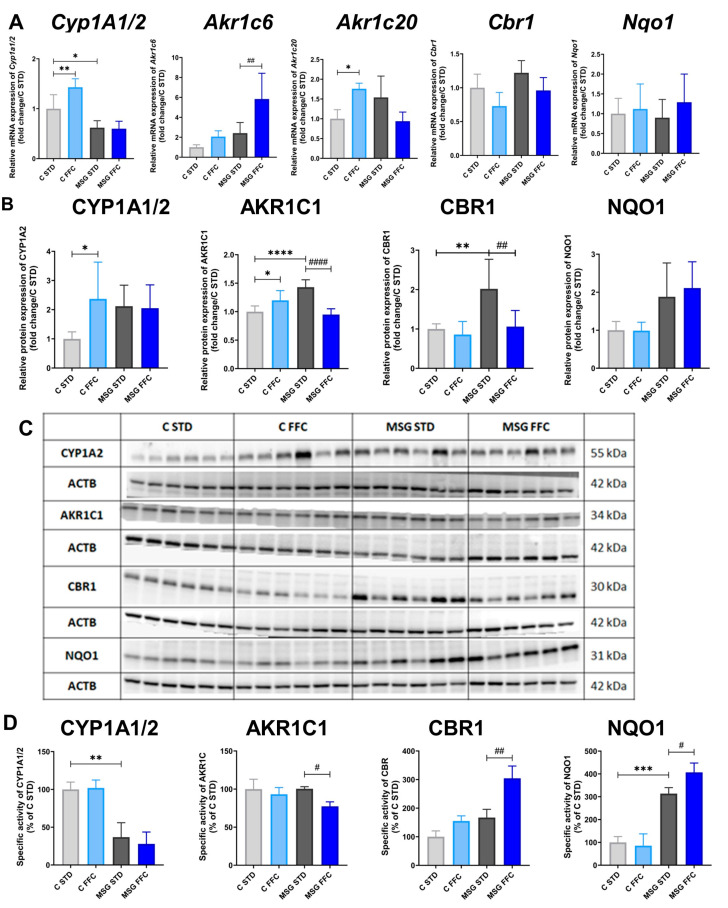
Cytochrome P450 1A1/2, aldo-keto reductase 1C, carbonyl reductase 1, and NAD(P)H:quinone oxidoreductase 1 expression and activity in the liver of MASLD models. (**A**) Relative quantification of *Cyp1a1/2*, *Akr1c*, *Cbr1*, and *Nqo1* mRNA expression. (**B**) Relative protein expression of CYP1A1/2, AKR1C, CBR1, and NQO1. (**C**) Representative Western blot images of CYP1A1/2, AKR1C, CBR1, and NQO1. (**D**) Relative quantification of specific activities of CYP1A1/2, AKR1C, CBR1, and NQO1. Statistical analyses were performed using one-way ANOVA with Bonferroni’s post hoc test. The *p*-value thresholds were defined as * (*p* < 0.05), ** (*p* < 0.01), *** (*p* < 0.001), and **** (*p* < 0.0001) compared to the C STD group, and as # (*p* < 0.05), ## (*p* < 0.01), and #### (*p* < 0.0001) compared to the MSG STD group, respectively.

In MSG mice, the specific activity of CYP1A as well as the mRNA level of *Cyp1A1/2* were decreased (Figure 7A,D), while CYP1A2 protein expression tended to increase (Figure 7B,C). Significant upregulation of mRNA and protein expressions of CYP1A1/2 was induced by the FFC diet in control but not in MSG mice (Figure 7A–C). In the case of NQO1, a threefold elevation in activity in the MSG STD group and its further augmentation by the FFC diet was observed (Figure 7D), but on the protein and mRNA levels, the changes were not significant (Figure 7A–C). The catalytic activity of CBR1 tended to increase in MSG mice compared to control ones; moreover, it was further elevated by the FFC diet both in control and MSG mice (Figure 7D). In MSG mice, CBR1 protein levels were substantially higher (Figure 7B,C), although on the mRNA level, the upregulation was not significant (Figure 7A). The AKR1C subfamily includes eight isoforms; in this study, only liver-specific isoforms AKR1C6 and AKR1C20 were tested. The FFC diet caused significant upregulation of AKR1C protein levels as well as *Akr1c20* mRNA levels (Figure 7A–C), but catalytic activity remained unchanged in control mice (Figure 7D). Moreover, substantial increases in protein levels were found in MSG STD compared to C STD mice (Figure 7A–C). Furthermore, FFC diet-fed MSG mice had markedly lower protein levels and specific activity of AKR1C1 in comparison with MSG STD mice (Figure 7B–D).

### 3.5. FFC and MSG Alter miRNA Signature and Expression of Epigenetic Regulatory Enzymes in Liver

**Figure 8:**
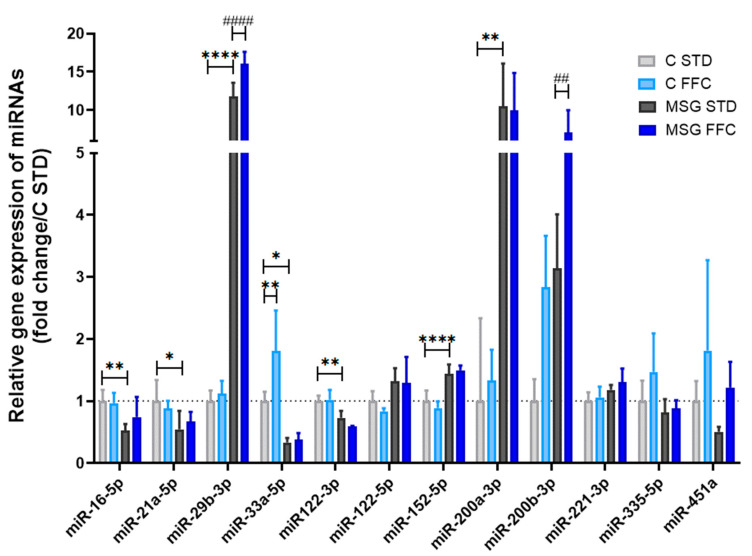
miRNA expression in liver. The relative microRNA expression was normalized to miR-93-5p and calculated using the 2^−ΔΔCt^ method. Results are presented as the mean ± SD (n = 6), with C STD set to 1. Statistical analyses were performed using one-way ANOVA with Bonferroni’s post hoc test. The *p*-value thresholds were defined as * (*p* < 0.05), ** (*p* < 0.01), and **** (*p* < 0.0001) compared to the C STD group, and as ## (*p* < 0.01) and #### (*p* < 0.0001) compared to the MSG STD group, respectively. The dash line indicates the expression level in C STD group.

Based on the literature review and using several miRNA-prediction algorithms [65,66,67,68], a panel of several miRNAs related to obesity, metabolic syndrome, and MASH was selected and tested. The obtained results are presented in Figure 8.

In the present study, the miRNA expression pattern was altered predominantly in the liver of MSG STD mice. These mice exerted considerably elevated levels of miR-29b-3p, miR-152-5p, miR-200a-3p, and miR-200b-3p, but downregulated levels of miR-16-5p, miR-21a-5p, miR-33a-5p, and miR-122-3p in comparison to C STD mice. In MSG FFC mice, two miRNAs displayed considerable upregulation (namely miR-29b-3p and miR-200b-3p) compared to MSG STD mice. In C FFC mice, miR-33a-5p and miR-200b-3p levels were markedly increased compared to the C STD group. In addition, levels of miR-122-5p, miR-221-3p, miR-335-5p, and miR-451a remained unchanged in all experimental groups.

Apart from miRNAs, modifications in epigenetic regulatory enzymes have been reported to contribute to the development of MASLD [30]. In this study, the gene expression of fifteen liver-expressed epigenetic regulatory enzymes was studied. The obtained results are shown in Figure S2.

Among DNMTs, mRNA levels of *Dnmt3a* were increased in FFC diet-fed mice, although significant change was observed only in the MSG FFC group. In mammals, the HDAC family comprises eighteen enzymes classified into four classes—eleven members of the classical HDAC family and seven members of the SIRT family [69]. In this study, a significant increase in *Hdac2* mRNA expression was found in MSG STD mice compared to C STD, while feeding the FFC diet resulted in its decreased levels in the MSG group. The levels of *Dnmt1*, *Hdac1*, *Hdac3*, *Hdac5*, *Hdac6*, *Hdac11*, *Sirt1*, *Sirt2*, *Sirt3*, *Hat1*, *Kat2b*, *Kat5*, and *Ep300* remained unchanged.

### 3.6. FFC and MSG Affect Expression of Several Genes Involved in Lipid Metabolism

To investigate whether metabolic processes were affected by MSG application or FFC diet feeding, gene expression of key genes implicated in hepatic lipid metabolism was determined. Obtained results are summarized in Table 2.

In MSG STD mice, mRNA levels of *Srebf2* were significantly upregulated, while those of *Gpam* were markedly downregulated compared to C STD mice. Feeding the FFC diet caused a considerable decrease in mRNA levels of *Srebf2* and a significant increase in *Cidec* mRNA levels both in control as well as MSG mice. Moreover, substantial upregulation in *Pparγ*, *Acaca*, and *Scd2* mRNA levels was found in MSG FFC mice in comparison to MSG STD mice.

## 4. Discussion

MASLD is closely associated with peripheral insulin resistance and intrahepatic oxidative stress, both of which contribute to liver injury and occur either by elevation of ROS and/or due to the decrease in antioxidant tissue levels [70]. In this work, we characterized alterations in the liver histology, detoxification enzymes, epigenetic regulation, and genes involved in lipid metabolism in three in vivo models of MASLD. Moreover, the present study highlights the importance of employing animal models to investigate the progression of MASLD to MASH and its association with metabolic syndrome. The use of obesogenic dietary models, such as the DIO model, which is fed the FFC diet, has been instrumental in mimicking the natural development of MASLD in mice, mirroring features of human MASLD such as steatosis, fibrosis, and inflammation. These models demonstrate increased body and liver weight, altered lipid and cholesterol profiles, elevated liver enzymes, and enhanced proinflammatory cytokines, closely resembling the human MASLD conditions [3,5,6,7,8].

The subcutaneous administration of MSG to newborn mice induces early-onset obesity with increased adiposity, and MSG-obese mice (even on STD) display characteristics of pre-diabetes, with hyperinsulinemia at nearly normal blood glucose levels [11,12,13]. In the present study, MSG mice were fed either the STD or FFC diet. The third model of MASLD is represented by MSG mice fed the FFC diet, which combines the effects of MSG application to newborn mice with the effects of the FFC diet in adulthood. The three MASLD models, i.e., control mice (without MSG application) on the FFC diet (group C FFC), MSG mice on STD (group MSG STD), and MSG mice on the FFC diet (group MSG FFC), were prepared, characterized, and compared. The control mice on STD represent the control group (group C STD).

The findings demonstrate that the mice fed with the FFC diet exhibited significantly higher body weight than their respective STD-fed counterparts at 7 months of age, while the MSG-treated mice on the STD had a significantly higher gWAT weight when compared to the control mice on the STD. The liver weight was significantly higher in both groups that were fed the FFC diet when compared to the STD-fed groups. However, no significant differences were observed in the OGTT results among the experimental groups, indicating that glucose tolerance did not vary significantly between the control and MSG mice or between the different diet groups. These data are consistent with previous studies [71,72,73].

In agreement with the prediabetic state, biochemical parameters, including plasma TAGs, FFA levels, and insulin concentration, did not differ significantly among experimental groups, except for elevated plasma cholesterol levels observed in the C FFC and MSG FFC groups. The FFC diet as well as MSG application increased liver TAG accumulation and plasma AST level due to the hepatocyte damage. The disrupted leptin signaling in MSG mice was confirmed by markedly higher plasma leptin levels in all MSG groups compared to corresponding non-MSG groups. These findings are consistent with previous studies [72,73,74].

A histological examination of the liver sections revealed notable liver steatosis and hepatocellular ballooning in both the C FFC and MSG FFC groups when compared to their respective controls, indicated by the massive accumulation of fat droplets. Additionally, extensive steatosis was observed in the MSG STD group compared to the C STD group. Hepatic fibrosis, assessed through Picrosirius Red staining of hepatic collagens and COL1A1 protein expression, was increased in the MSG FFC group compared to the MSG STD group, while the C FFC group did not show increased fibrosis compared to the C STD group. These findings are consistent with those reported in the literature [71,74,75]. The feeding of mice with a Western diet together with fructose and glucose in their drinking water [76] resulted in the development of liver steatosis and fibrosis. However, our previous study [77] did not reveal the presence of fibrosis in a mouse model fed a high-fat diet with fructose and sucrose in its drinking water. Our recent study, which combined the leptin resistance of the MSG model of early-onset obesity with FFC feeding, resulted in the development of strong steatosis with ballooning, increased collagen, and fibrosis.

The accumulation of TAGs in the liver, a hallmark of MASLD [78], was confirmed in our study through the assessment of hepatic TAG content and changes in mRNA expression of the genes involved in lipid metabolism. In MSG STD mice, the relative expressions of transcription factors *Pparγ* and *Srebf2* and their target genes were augmented, which is in concordance with previously published results [79,80]. The FFC diet caused elevated levels of *Pparγ*, genes involved in *de novo* lipogenesis (e.g., *Acaca*, *Scd2*), and *Cidec* in both control and MSG mice. On the other hand, the relative expression of genes involved in cholesterol synthesis (e.g., *Srebf2*, *Hmgcr*) was reduced by FFC feeding in both groups of mice. Similar results were obtained in transgenic S-mice fed a high-fat, high-sucrose diet [81].

Our work then focused on monitoring alterations in the antioxidant and drug-metabolizing systems of the liver, in addition to the epigenetic regulators. Liver injury observed in MASLD is at least partially driven by oxidative stress [82] and is accompanied by oxidative damage to biomolecules [83,84,85]. The harmful effects of ROS are counteracted by the activity of the antioxidant defense system composed of non-enzymatic (e.g., GSH) and enzymatic antioxidants (e.g., SOD, CAT, GPx, GR) [21]. Additionally, certain isoforms of GSTs exert selenium-independent GSH-peroxidase activity and can therefore be considered a part of the antioxidant defense system [86]. In human and animal studies reported MASLD-associated changes in the antioxidant enzymes activity and/or expression are inconsistent [82], which can be attributed to differences in experimental diet composition, animal strain susceptibility, disease stage, and genetic polymorphisms. In our study, MSG STD mice exhibited increased GPx activity while overall GST activity was reduced. Furthermore, SOD1 and CAT activities were elevated in both FFC-fed groups of mice. This elevation in catalytic activities may indicate an adaptive response to augmented ROS generation during the initial phases of MASLD [23]. The decreased GST activity could be ascribed to the diminished mRNA and protein expression of GSTP-class isoforms [46]. Moreover, GSTs conjugate GSH to lipid peroxidation products (e.g., 4-hydroxynonenal) and exogenous unsaturated aldehydes (e.g., acrolein). Therefore, the downregulation of GST isoforms and/or decrease in GST activity observed in mice fed a high-fat diet (HFD), obese humans, and MASLD patients could lead to the reduced capacity to manage electrophilic compounds, their accumulation, and increased protein carbonylation by lipid peroxidation products [23,87,88]. In our study, FFC feeding caused upregulation of relative *Gpx7* expression, which is presumably attributable to the increased unfolded protein response as a consequence of hepatic lipid overload [19]. Moreover, GPx7 was highly overexpressed in a mouse model of liver MASH fibrosis, and in liver LX-2 cells, its overexpression ameliorated ROS formation and decreased the expression of pro-fibrotic and proinflammatory genes [89]. The observed discrepancy between the gene expression levels and enzymatic activities of the antioxidant enzymes (e.g., SOD1 and CAT) can be attributed to both post-transcriptional and post-translational regulatory mechanisms. Post-transcriptional regulation affects mRNA processing (including alternative splicing), stabilization or degradation, transport, and translation into proteins, and post-translational regulation governs protein modifications, activation, degradation, and proper folding to achieve functionality [90]. While gene expression levels reflect only transcriptional activity, enzyme activity is influenced by additional factors such as protein stability, post-translational modifications, and interactions with cofactors. Moreover, cellular conditions like oxidative stress can activate pre-existing enzyme pools, increasing activity without a corresponding rise in mRNA levels [91].

The liver is the primary organ responsible for the metabolism and elimination of a variety of drugs. Abundant evidence underscores that MASLD/MASH patients are more prone to drug–drug interactions and adverse drug reactions, primarily due to significant alterations in DMEs. These enzymatic disruptions may modify the clearance rate of therapeutic drugs, giving rise to impaired drug efficacy, therapeutic failure, or increased drug toxicity [26,92]. Despite a decrease in CBR1 protein expression, its specific activity exhibited an elevation in the MSG FFC group, indicating a posttranslational mechanism of its regulation. This enzyme participates in the metabolism of a number of endogenous compounds (e.g., isatin) as well as drugs containing a carbonyl group (e.g., anthracyclines and warfarin) [93]. Moreover, CBR1 is involved in the regulation of tissue glucocorticoid metabolism, glucose homeostasis, and the reduction in lipid peroxidation products [94,95], and may contribute to the cardiometabolic complications of obesity [96]. In our study, the catalytic activity of NQO1 was elevated in MSG STD mice and further increased in MSG FFC mice [97]. In patients with MASLD, *Nqo1* mRNA and protein expression as well as activity tend to increase with the progression of the disease [23]. A similar elevation in the catalytic activity and/or expression of NQO1 was observed in methionine- and choline-deficient diet-fed MASLD rats [98]. Induction of NQO1 could therefore contribute to greater protection of an organism against quinone redox cycling induced by oxidative stress or pharmacological medication.

In order to gain further insight into the mechanisms involved in MASLD pathogenesis, we focused on the MSG and/or FFC feeding-induced changes in the gene expression of selected miRNAs and epigenetic regulatory enzymes in liver tissue. In our experiments, the expression of liver miRNAs was deregulated mainly in the MSG STD group, in which miR-29b-3p, miR-200a-3p, and miR-200b-3p belonged to the most upregulated, while miR-16-5p, miR-21a-5p, miR-33a-5p, and miR-122-3p were among the most downregulated miRNAs. Moreover, the relative expression of miR-200b was further increased by the FFC diet in both control and MSG mice. In our previous study, we observed an upregulation of hepatic miR-200b in a high-fat diet with fructose in C57/BL6 male mice, although the extent of this upregulation was less pronounced than in the current study [77]. This miRNA regulates key cellular processes such as epithelial–mesenchymal transition, fibrosis, and inflammation by targeting specific mRNAs and modulating gene expression within these pathways [99]. In the context of MASLD, miR-200b-3p contributes to disease pathogenesis by modulating immune responses; its elevated levels have been detected in both fatty liver [100] and fibrotic liver samples [101], confirming its involvement in disease progression. Given its stability in circulation, miR-200b-3p holds promise as a non-invasive biomarker for diagnosing and monitoring MASLD. Therapeutically, modulating miR-200b-3p levels could reduce inflammation and fibrosis, potentially slowing MASLD progression. Differences in the expression of some hepatic miRNAs (e.g., miR-200b) may cause different susceptibility of individual mouse strains to the development of diet-induced MASLD/MASH [38,102]. In human steatotic livers, the expression of miR-200 family members (miR-200a/b/c) was upregulated in comparison to the livers of healthy donors [103]. Moreover, its overexpression was related to the grade of liver fibrosis in human liver specimens [104]. Employing prediction algorithms, we discerned that these upregulated miRNAs regulate the transcription of *Ppara*. Among experimentally verified target genes of the miR-29 family belong de novo DNA methyltransferases *Dnmt3a* and *Dnmt3b* [105]. However, multiple-to-multiple interactions between miRNAs and their target genes were observed in MASLD [106]. Regarding epigenetic regulatory enzymes, changes in their mRNA expressions were mostly of a lower extent, which would likely lack biological relevance.

## 5. Conclusions

Based on our results, MSG mice fed the FFC diet could be considered a suitable model of MASLD characterized by both liver steatosis and fibrosis. In addition, our study has revealed substantial modifications in hepatic lipid metabolism, epigenetic regulatory factors, and expressions and activities of various detoxification enzymes (namely SOD1, CAT, and CBR1) in MASLD mice compared to control mice. miR-200b-3p, highly significantly upregulated in both FFC groups, could be considered as a potential diagnostic marker of MASLD; however, further research is needed.

The limitation of the study is the use of outbred NMRI and not inbred strain. The reason for this was both practical, as NMRI pups are more robust, easier to handle and treat, and ethical, as nearly all MGS-treated pups—unlike C57BL/6 ones—easily survived the treatment. Moreover, MSG NMRI males reach a very high HOMA index in adulthood, which is a favorable parameter for MASLD development.

## Figures and Tables

**Table 1:** Analysis of body weight, liver weight, and plasma biochemical parameters in MASLD mouse models. At the end of the experiment, body weight, nasoanal length, gonadal white adipose tissue, liver weight, hepatic TAG content, and plasma glucose, AST, TAGs, FFAs, cholesterol, insulin, and leptin levels were measured.

	C STD	C FFC	MSG STD	MSG FFC
BW (g)	45.79 ± 1.88	54.78 ± 2.99 ****	46.60 ± 1.41	56.86 ± 4.133 ####
NA length (cm)	11.22 ± 0.28	11.85 ± 0.37 *	10.62 ± 0.42 ×	10.88 ± 0.31
Lee index	319.05 ± 8.58	320.68 ± 11.73	339.30 ± 11.88 ×	353.31 ± 10.03
gWAT (g)	0.56 ± 0.28	3.00 ± 0.77 ****	1.82 ± 0.83 ×	2.18 ± 0.60
Liver weight (g)	1.89 ± 0.20	2.88 ± 0.48 *	1.66 ± 0.16	3.03 ± 1.03 ##
Hepatic TAG (mg/g)	6.75 ± 3.59	18.34 ± 11.73 *	9.64 ± 2.82	19.31 ± 7.80
Glucose (mmol/L)	8.51 ± 2.11	8.63 ± 2.66	6.18 ± 0.72	6.02 ± 1.99
AST (IU/L)	198.62 ± 126.94	532.72 ± 488.68	308.83 ± 16.04	593.56 ± 327.09
TAG (mmol/L)	105.42 ± 43.32	117.81 ± 22.49	126.37 ± 34.11	107.29 ± 28.36
CHOL (mmol/L)	90.61 ± 12.26	147.53 ± 57.76	126.02 ± 33.65	181.90 ± 33.25
FFA (mmol/L)	114.15 ± 32.49	107.44 ± 28.42	107.06 ± 40.70	84.89 ± 27.07
Leptin (ng/mL)	118.18 ± 96.95	1174.19 ± 542.58	2522.51 ± 1472.15 ××	4749.53 ± 2882.7 #
Insulin (ng/mL)	0.19 ± 0.14	0.36 ± 0.30	0.31 ± 0.31	0.83 ± 0.61

Results are presented as the mean ± SD (n = 6, for AST n = 5). Statistical analyses were performed using one-way ANOVA with Bonferroni´s post hoc test. The *p*-value thresholds were defined as * (*p* < 0.05) and **** (*p* < 0.0001) for C FFC compared to C STD group; # (*p* < 0.05), ## (*p* < 0.01), and #### (*p* < 0.0001) for MSG FFC compared to MSG STD group; and × (*p* < 0.05) and ×× (*p* < 0.01) for MSG STD compared to C STD, respectively.

**Table 2:** qPCR analysis of selected genes involved in lipid metabolism.

Gene	C STD	C FFC	MSG STD	MSG FFC
*Ppara*	1.00 ± 0.33	1.23 ± 0.49	1.08 ± 0.20	1.00 ± 0.46
*Pparg*	1.00 ± 0.26	2.33 ± 0.73	1.70 ± 0.61	4.23 ± 2.47 ^##^
*Acaca*	1.00 ± 0.16	1.24 ± 0.32	1.25 ± 0.22	1.84 ± 0.21 ^##^
*Fasn*	1.00 ± 0.29	0.94 ± 0.68	1.76 ± 0.71	1.38 ± 1.16
*Scd1*	1.00 ± 0.47	3.92 ± 2.74	3.45 ± 1.53	6.56 ± 3.93
*Scd2*	1.00 ± 0.55	1.30 ± 0.40	0.72 ± 0.12	2.69 ± 0.83 ^####^
*Srebf2*	1.00 ± 0.17	0.55 ± 0.13 **	1.68 ± 0.26 ^××××^	0.84 ± 0.16 ^####^
*Hmgcr*	1.00 ± 0.20	0.62 ± 0.26	1.75 ± 0.92	0.92 ± 0.34
*Gpam*	1.00 ± 0.17	0.77 ± 0.27	0.31 ± 0.21 ^××××^	0.19 ± 0.10
*Cidec*	1.00 ± 1.18	4.51 ± 1.83 **	0.82 ± 0.52	6.27 ± 3.00 ^###^
*Plin2*	1.00 ± 0.35	1.18 ± 0.33	1.34 ± 0.63	1.64 ± 0.32
*Plin5*	1.00 ± 0.33	0.90 ± 0.26	1.57 ± 0.67	0.98 ± 0.46

The mRNA expression was normalized to the geomean of two reference genes, *Gapdh* and *Rplp0*, and calculated using the 2^−ΔΔCt^ method. Results are presented as the mean ± SD (n = 6), with C STD set to 1. Statistical analyses were performed using one-way ANOVA with Bonferroni’s post hoc test. The *p*-value thresholds were defined as ** (*p* < 0.01) for C FFC compared to the C STD group; ^##^ (*p* < 0.01), ^###^ (*p* < 0.001), and ^####^ (*p* < 0.0001) for MSG FFC compared to the MSG STD group; ^××××^ (*p* < 0.0001) for MSG STD compared to C STD, respectively.

## Data Availability

All data obtained from the study can be found in the article/Supplementary Material; further information can be obtained by contacting the corresponding author.

## Supplementary Materials

The following supporting information can be downloaded at: https://www.mdpi.com/article/10.3390/antiox13111371/s1, Figure S1: mRNA expression and activity of glutathione reductase; Figure S2: mRNA expression of epigenetic regulatory enzymes. Results are presented as the mean ± SD (n = 6), with C STD set to 1. Statistical analyses were per-formed using one-way ANOVA with Bonferroni’s post hoc test. The *p*-value thresholds were defined as * (*p* < 0.05) for C FFC compared to the C STD group; # (*p* < 0.05) for MSG FFC compared to the MSG STD group.; Table S1: List of primers; Table S2: List of primary and secondary antibodies for Western blot.
