# Supplementary material for: Metabolic Dysfunction-Associated Steatotic Liver Disease Is Accompanied by Increased Activities of Superoxide Dismutase, Catalase, and Carbonyl Reductase 1 and Levels of miR-200b-3p in Mouse Models
Source: Antioxidants (Basel). 2024 Nov 9;13(11):1371. doi: 10.3390/antiox13111371 (PMC11591148; doi:10.3390/antiox13111371)
Supplement: Supplementary file 1 [file antioxidants-13-01371-s001.zip › antioxidants-3256914-supplementary.pdf]

Supplementary figures

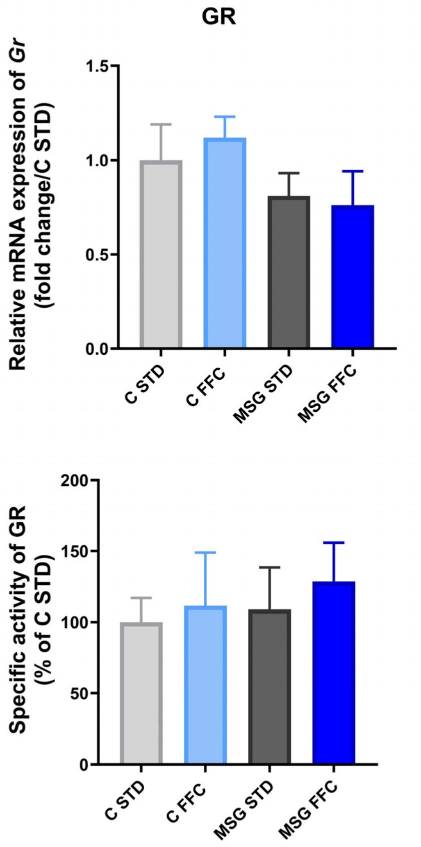

Figure S1 mRNA expression and activity of glutathione reductase. Statistical analyses were performed using one-way ANOVA with Bonferroni post-hoc test.

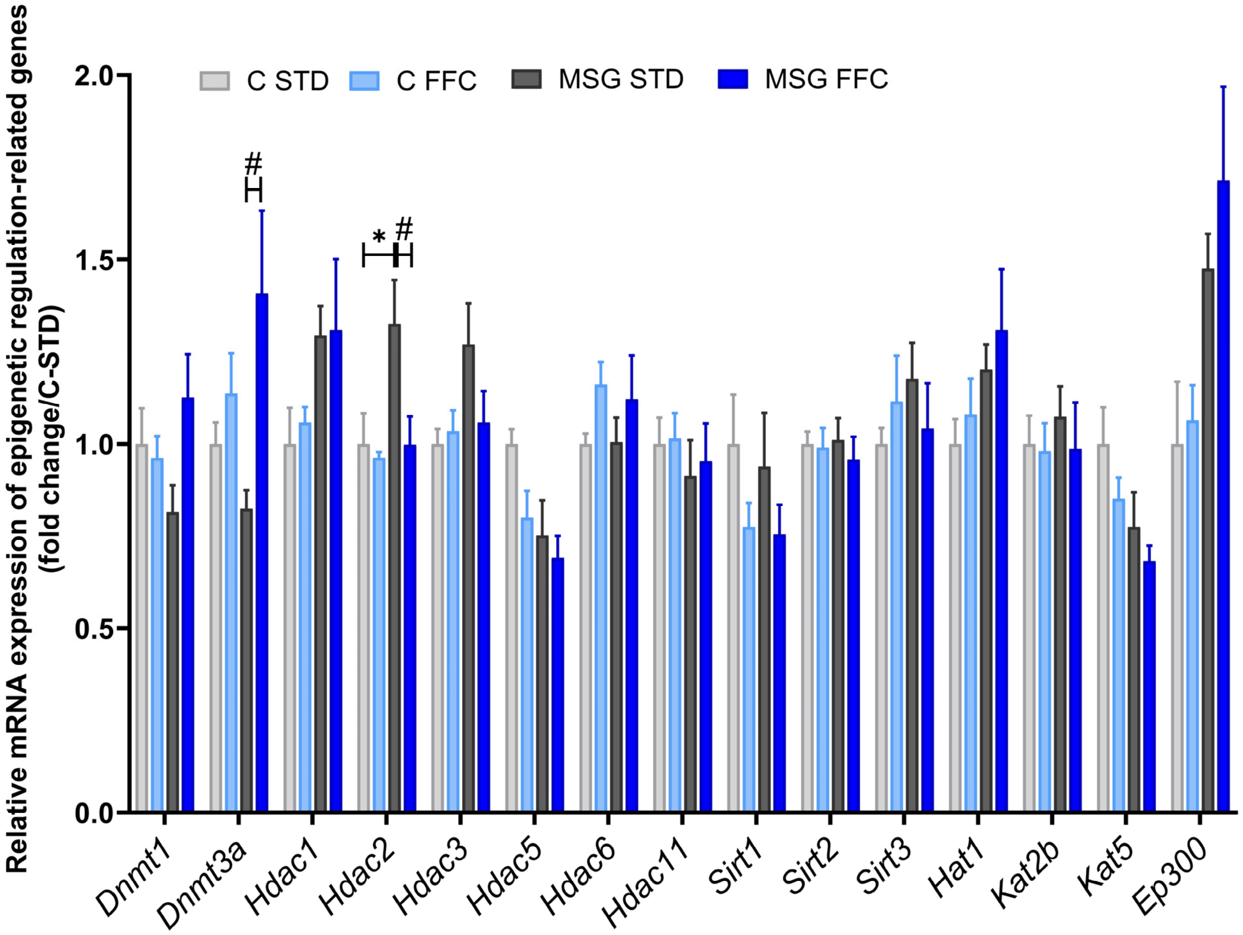

Figure S2 mRNA expression of epigenetic regulatory enzymes. The relative mRNA expression was normalized to geometric mean of GAPDH and RPLP0 and calculated using $2^{-\Delta\Delta Ct}$ method. Results are presented as the mean ± SD (n=6), with C STD set to 1. Statistical analyses were performed using one-way ANOVA with Bonferroni post-hoc test. The *p*-value thresholds were defined as * ($p < 0.05$) for C FFC compared to the C STD group; # ($p < 0.05$) for MSG FFC compared to the MSG STD group.

Supplementary tables:

Table S1 List of primers

	Gene	NCBI Accession No.	Forward primer	Reverse primer	Amplicon size
Reference Genes	***Gapdh***	NM_008084	5´-AGGTCGGTGTGAACGGATTTG-3´	5´-TGTAGACCATGTAGTTGAGGTCA-3´	123
	Rplp0	NM_007475	5´-GATGGGCAACTGTACCTGACTG-3´	5´-CTGGGCTCCTCTTGGAATG-3´	136
Genes Involved in Lipid Metabolism	***Ppara***	NM_011144	5´-GAACCGGAACAAATGCCAGT-3´	5´-TTGTGTGACATCCCGACAGA-3´	63
	Pparg	NM_001127330	5´-AGACCCAGCTCTACAACAGG-3´	5´-ACAGACTCGGCACTCAATGG-3´	71
	Acaca	NM_133360	5´-TTCGCCATAACCAAGTAGAGTC-3´	5´-AGGATGAGTTTCTGCAGGTTC-3´	91
	Fasn	NM_007988	5´-CTGAAGCCGAACACCTCTGT-3´	5´-GCCTCAGAGCGACAATATCC-3´	107
	Scd1	NM_009127	5´-CAGGTTTCCAAGCGCAGTT-3´	5´-GATCTCTTGGAGCATGTGGG-3´	136
	Scd2	NM_009128	5´-AGTATTGCCACCCAGATGCT-3´	5´-AGAAGGAAGAGCTGTCGACT-3´	100
	Srebf2	NM_033218	5´-ACTCCTTCTGTCACTGTGAGA-3´	5´-CAGAAGTAGTGCCGCTGAC-3´	72
	Hmgcr	NM_008255	5´-CAGAACCTACGGCAGCTTG-3´	5´-AGGCCAGCAATACCCAGAAT-3´	50
Genes Related to Epigenetic Regulation	***Dnmt1***	NM_001199432	5´-AACCCCAGATGTTGACCAGT-3´	5´-GGAAGTGAACCTATGCATGGG-3´	98
	Dnmt3a	NM_153743	5´-CCATGGGCGTTAGTGACAAG-3´	5´-TGCAGCAGACACTTCTTTGG-3´	86
	Hdac1	NM_008228	5´-GATCCCTAATGAGCTGCCCT-3´	5´-TGTTGGAAGGGCTGATGTGA-3´	80
	Hdac2	NM_008229	5´-AGGTGAAGGAGGTCGTAGGA-3´	5´-TGTCCTCTGTCTCCTTCTTGT-3´	89
	Hdac3	NM_010411	5´-ACTTCGAGTACTTTGCCCCA-3´	5´-TTCTGATTCTCGATGCGGGT-3´	67
	Hdac5	NM_001077696	5´-CACGCTTCTTTGGACCAGAG-3´	5´-GGAAAGTCATCACGGCTGTC-3´	107
	Hdac6	NM_001411604	5´-CATGCTGGAGTCCTGTGTTG-3´	5´-AGTAGATGGAAGTCTGGGCG-3´	53
	Hdac11	NM_144919	5´-CGCACAGCCCGTATTATCG-3´	5´-CCCAGGTCATGCAAGTTGAG-3´	50
	Sirt1	NM_001159589	5´-ACATCTCATGATTGGCACCG-3´	5´-GCCACAGCGTCATATCATCC-3´	95
	Sirt2	NM_001122766	5´-AGAATACACGATGGGCTGGA-3´	5´-CTCTGACACTGCTCACACCT-3´	72
	Sirt3	NM_001177804	5´-GGCCTCTACAGCAACCTTCA-3´	5´-AAGATGGCTTCAGGGTACGG-3´	53
	Hat1	NM_026115	5´-TGGATGATGAAAGATGGCACT-3´	5´-CGGGTTTTGTCTGGGTACAC-3´	124
	Kat2b	NM_001190846	5´-AAAGAGCCCAAAGACCCTGA-3´	5´-AGGATGTTCTTGAGGGTGCT-3´	50
	Kat5	NM_001362370	5´-TGTCATCTCCACACTGCAGT-3´	5´-GCTCATGCCCATCCACGAT-3´	95
	Ep300	NM_177821	5´-AGATTCCACCACAACCCCAG-3´	5´-TGGCTGGCTTTGTTGATTCT-3´	50
Genes Encoding Antioxidant enzymes	***Sod1***	NM_011434	5´-AACCAGTTGTGTTGTCAGGAC-3´	5´-CCACCATGTTTCTTAGAGTGAGG-3´	139
	Cat	NM_009804	5´-AGCGACCAGATGAAGCAGTG-3´	5´-TCCGCTCTCTGTCAAAGTGTG-3´	181
	Gr	NM_010344	5´-AGCAGTGCACTCGGAATTCA-3´	5´-CGAATGTTGCATAGCCGTGG-3´	182
	Gpx1	NM_008160	5´-GAATGCCTTGCCAACACCC-3´	5´-GTACTTGGGGTCGGTCATGA-3´	58
	Gpx4	NM_008162	5´-TTGATAAGAACGGCTGCGTG-3´	5´-CACACTTGTAGGGCTAGCTAGA-3´	103
	Gpx7	NM_024198	5´-TTTGGCCAACAGGAACCAGA-3´	5´-GGCGGGCAAAGTTCTCAATC-3´	52
	Gsta1/2	NM_008181/NM_008182	5´-GATTGGGCAATTGGTATTATGTC-3´	5´-CCTGTTGCCCACAAGGTAGT-3´	142
	Gsta3	NM_010356	5´-GACCTGGCAAGGTTACGAAG-3´	5´-TATCTCCAGATCCGCCACTC-3´	195
	Gsta4	NM_010357	5´-CCTCGCTGCCAAGTACAACT-3´	5´-TTGCCAACGAGAAAAGCCTC-3´	231
	Gstm1	NM_010358	5´-AATTGGGATTGGTGCAGGGT-3´	5´-ACTGACCTGTGTGTTTGGAGT-3´	101
	Gstm3	NM_010359	5´-GCTCATGATAGTCTGCTGCAG-3´	5´-GCTTCATTTTCTCAGGGATGGC-3´	80
	Gstp	NM_013541	5´-AGCCTTTTGAGACCCTGCTG-3´	5´-CGGCAAAGGAGATCTGGTCA-3´	75
Genes Encoding phase I DMEs	***Cyp1a1/2***	NM_009992/NM_009993	5´-TCACTGACACGGCTGAGT-3´	5´-GTCGGCCCTTGAAGTCATC-3´	136
	Cyp2c29	NM_007815	5´-TGTCCTGGAAGTCATCACAAA-3´	5´-GGGGTTTGTAACATCCAGTGA-3´	105
	Cyp3a11	NM_007818	5´-CCTCTGAAATTAAGCAGACAAGGA-3´	5´-CGATGTTCTTAGACACTGCCTT-3´	149
	Cyp3a13	NM_007819	5´-AAAGGCCATCTCTATCTCTGAGA-3´	5´-CACCAACACATCTGTAAACTGGTTA-3´	127
	Cyp3a25	NM_019792	5´-TTCACTGTCCAGCCTTGTGAA-3´	5´-AAATTGGTTCCCTGCTGATCTTC-3´	61
	Fmo1	NM_010231	5´-TCCTGACCTCTATCAACGCAAAA-3´	5´-CTTGTATGGTTCGAGTTTTGGTGA-3´	201
	Fmo5	NM_001161765	5´-ACCTGCTACTCTCTTCTCGGA-3´	5´-CCACAGATACGTGACAAGTAGT-3´	49
	Akr1a	NM_021473	5´-AGCCTGGTCAGGTGAAAGC-3´	5´-GGCCTCCCCAATCTCAGTT-3´	104
	Akr1c6	NM_030611	5´-TGTCACTGCATTGGAAGAGTG-3´	5´-TCAGAAGCTTGGATTAGGGTGA-3´	80
	Akr1c20	NM_054080	5´-GGCAAAGCTAAGAGTTGCAGA-3´	5´-TGGTAAACGTCACATGGGTTCA-3´	100
	Cbr1	NM_007620	5´-GCTGCTCCCTCTAATAAAACCC-3´	5´-CCTCTGTGATGGTCTCGCTTC-3´	125
	Nqo1	NM_008706	5´-GTCCATTCCAGCTGACAACC-3´	5´-TCCTTTTCCCATCCTCGTGG-3´	142

	miRNA	miRBase Accession No.	RT primer	Forward primer
reference miRNA	**miR-93-5p**	MIMAT0000540	5´-gtc tcc tct g gt gca ggg tcc gag gta ttc gc a c ca gag gag ac cta cct-3´	5´-GTC AAT CAA AGT GCT GTT CGT G-3´
miRNAs Associated with Lipid Metabolism and Obesity	**miR-16-5p**	MIMAT0000527	5´-gtc tcc tct g gt gca ggg tcc gag gta ttc gc a c ca gag gag acc gcc aa-3´	5´-ACA GCC TAG CAG CAC GTA AAT-3´
	**miR-21a-5p**	MIMAT0000530	5´-gtc tcc tct g gt gca ggg tcc gag gta ttc gc a c ca gag gag act caa ca-3´	5´-GGA TGA CGT AGC TTA TCA GAC TG-3´
	**miR-29b-3p**	MIMAT0000127	5´-gtc tcc tct g gt gca ggg tcc gag gta ttc gc a c ca gag gag ac aac act-3´	5´-GCC GTT AGC ACC ATT TGA AAT C-3´
	**miR-33a-5p**	MIMAT0000667	5´-gtc tcc tct g gt gca ggg tcc gag gta ttc gc a c ca gag gag ac tgc aat-3´	5´-AGG TTC GGT GCA TTG TAG TTG-3´
	**miR-122-3p**	MIMAT0017005	5´-gtc tcc tct g gt gca ggg tcc gag gta ttc gc a c ca gag gag ac tat tta-3´	5´-GAG CTC AAC GCC ATT ATC ACA-3´
	**miR-122-5p**	MIMAT0000246	5´-gtc tcc tct ggt gca ggg tcc gag gta ttc gca cca gag gag acc aaa ca-3´	5´-GGC TGT GGA GTG TGA CAA TG-3´
	**miR-152-3p**	MIMAT0000162	5´-gtc tcc tct g gt gca ggg tcc gag gta ttc gc a c ca gag gag ac cca agt-3´	5´-CGA CGT TCA GTG CAT GAC AG-3´
	**miR-200a-3p**	MIMAT0000519	5´-gtc tcc tct g gt gca ggg tcc gag gta ttc gc a c ca gag gag ac aca tcg-3´	5´-GCG TCC TAA CAC TGT CTG GT-3´
	**miR-200b-3p**	MIMAT0000233	5´-gtc tcc tct g gt gca ggg tcc gag gta ttc gc a c ca gag gag ac tca tca-3´	5´-GGT GCT AAT ACT GCC TGG TA-3´
	**miR-221-3p**	MIMAT0000669	5´-gtc tcc tct g gt gca ggg tcc gag gta ttc gc a c ca gag gag ac aaa ccc-3´	5´-CTG CCA GCT ACA TTG TCT GC-3´
	**miR-335-5p**	MIMAT0000766	5´-gtc tcc tct g gt gca ggg tcc gag gta ttc gc a c ca gag gag ac aca ttt-3´	5´-ACC ACC ATC AAG AGC AAT AAC G-3´
	**miR-451a**	MIMAT0001632	5´-gtc tcc tct g gt gca ggg tcc gag gta ttc gc a c ca gag gag ac aac tca-3´	5´-CGA CCG AAA CCG TTA CCA TT-3´

	sequence
**universal reverse primer**	5´-GTG CAG GGT CCG AGG TAT TC-3´

Table S2 List of primary and secondary antibodies for western blot

Primary antibodies	Type	Dilution	Cat. No.	Manufacturer
anti-AKR1C1	rabbit polyclonal	1 : 10,000	ab192785	Abcam, Cambridge, UK
anti-β-actin	rabbit polyclonal	1 : 10,000	ab8227	
anti-CAT	rabbit polyclonal	1 : 5,000	ab16731	
anti-CBR1	rabbit recombinant monoclonal	1 : 10,000	ab174852	
anti-GPx4	rabbit recombinant monoclonal	1 : 10,000	ab125066	
anti-GPx7	rabbit polyclonal	1 : 2,000	ab96257	
anti-GSTμ	goat polyclonal	1 : 2,000	ab53942	
anti-GSTπ	rabbit recombinant monoclonal	1 : 10,000	ab138491	
anti-NQO1	rabbit recombinant monoclonal	1 : 2,000	ab80588	
anti-SOD1	rabbit polyclonal	1 : 10,000	ab16831	
anti-CYP1A2	mouse monoclonal	1 : 5,000	#14719	Cell Signaling Technology,
anti-COL1A1	rabbit monoclonal	1 : 1,000	#91144	Danvers, MA, USA

Secondary antibodies	Type	Dilution	Cat. No.	Manufacturer
anti-rabbit	goat polyclonal	1 : 10,000	ab97051	Abcam, Cambridge, UK
anti-goat	bovine polyclonal	1 : 10,000	sc-2350	Santa Cruz Biotechnology,
anti-mouse	bovine polyclonal	1 : 10,000	sc-2371	Dallas, TX, USA
